# Inter- and intra-city comparisons of PM_2.5_ concentration changes under COVID-19 social distancing in seven major cities of South Korea

**DOI:** 10.1007/s11869-021-01006-w

**Published:** 2021-03-11

**Authors:** Kyung-Hwan Kwak, Beom-Soon Han, Kyeongjoo Park, Sungju Moon, Han-Gyul Jin, Seung-Bu Park, Jong-Jin Baik

**Affiliations:** 1 School of Natural Resources and Environmental Science, Kangwon National University, Chuncheon, South Korea; 2 Department of Biological and Environmental Engineering, Semyung University, Jecheon, South Korea; 3 School of Earth and Environmental Sciences, Seoul National University, Seoul, South Korea; 4 School of Environmental Engineering, University of Seoul, Seoul, South Korea

**Keywords:** COVID-19, PM_2.5_, Urban air quality, Inter-city variability, Intra-city variability, South Korea

## Abstract

The COVID-19 pandemic has prompted governments around the world to impose mitigation strategies of unprecedented scales, typically involving some form of restrictions on social activities and transportation. The South Korean government has been recommending a collection of guidelines now known as social distancing, leading to reduced human activities. This study analyzes changes in the concentrations of fine particulate matter (PM_2.5_) during the 30-day periods before and since the start of social distancing on 29 February 2020 using measurement data from air quality monitoring stations at various locations of the seven major cities of South Korea, namely, Seoul, Busan, Incheon, Daegu, Daejeon, Gwangju, and Ulsan. All seven cities experienced decreased levels of PM_2.5_ concentration by up to 25% and smaller fluctuations during the period of social distancing. Inter-city comparisons show that the PM_2.5_ concentration changes are positively correlated with the city-wide PM_2.5_ emission fractions for mobile sources and negatively correlated with the city-wide PM_2.5_ emission fractions for combustion and industrial process sources. In addition, the meteorological influences favorable for transboundary pollutant transport have weakened during the period under COVID-19 social distancing. Intra-city comparisons show that decreases in the intra-city variability of PM_2.5_ concentration were larger in coastal cities than in inland cities. Comparisons between the inter- and intra-city variabilities in the PM_2.5_ concentration changes under social distancing highlight the importance of taking into account intra-city variabilities in addition to inter-city variabilities.

## Introduction

Managing the coronavirus disease 2019 (COVID-19) pandemic has now become a top priority for national and city governments worldwide. Prevention measures typically involve restrictions on human mobility such as lockdown and social distancing. Such societal changes during the COVID-19 pandemic can be more prominent in densely populated cities. A positive effect of lockdown and social distancing is general improvements in urban air quality, which are attributed to reductions of air pollutant emissions in transportation and industrial sectors (Le et al. 2020; He et al. 2020; Wang et al. 2020). While the COVID-19 pandemic has led to an increase in mortality directly from the infectious disease itself, it was reported that in some cases there has actually been a decrease in air pollution-related mortality owing to the improved air quality (Zambrano-Monserrate et al. 2020; Son et al. 2020). The impacts of COVID-19 mitigation measures on urban air quality has thus emerged as an important issue in environmental research and public health (Dutheil et al. 2020; Lokhandwala and Gautam 2020).

The impacts of COVID-19 on urban air quality, especially regarding primary pollutants (e.g., particulate matter (PM), nitrogen oxides (NO_x_), and carbon monoxide (CO)), have been reported in many cities where city-wide lockdowns were imposed (Bao and Zhang 2020; Mahato and Ghosh 2020; Zhu et al. 2020). Chauhan and Singh (2020) analyzed the concentration of PM_2.5_ (particulate matter with an aerodynamic diameter smaller than 2.5 μm) in nine major cities around the world (New York, Los Angeles, Zaragoza, Rome, Dubai, Delhi, Mumbai, Beijing, and Shanghai) and showed the decline in PM_2.5_ concentration in association with reductions in human activity levels due to COVID-19. Rodríguez-Urrego and Rodríguez-Urrego (2020) reported over 40% reductions in PM_2.5_ concentration during the quarantine period in Bogotá, Kubait City, Delhi, and Tehran, some of the most highly polluted cities in the world. General improvements in air quality between the periods before and after social distancing have also been reported for Seoul, South Korea (Han and Hong 2020; Ju et al. 2021).

The impacts of COVID-19 mitigation measures on urban air quality show both city-to-city variabilities and variabilities within individual cities (Bao and Zhang 2020; Berman and Ebisu 2020). Hereafter, the term “inter-city” is used to refer to any such comparisons among different cities and the term “intra-city” to refer to comparisons among different localities within a city. The assessments of air quality changes under social distancing in this study adopt both the inter- and intra-city points of view.

Inter-city comparisons of the impacts of COVID-19 on air quality have been made for various groups of cities around the world. Bao and Zhang (2020) analyzed the diverse effects of travel restrictions on reduction in air pollution using the differences in air quality index (AQI) among 44 cities in northern China. Briz-Redón et al. (2021) compared air quality changes in 11 cities of Spain during the period of COVID-19 lockdowns and showed that reductions of NO_2_ and PM_10_ concentrations were considerable in large cities such as Madrid, Barcelona, Valencia, and Sevila. Such inter-city variabilities are subject to influence by various factors such as changes in traffic-related emissions (Xiang et al. 2020; Chen et al. 2021) and meteorological conditions including air temperature, wind speed, and solar insolation (Ordόñez et al. 2020).

When it comes to the impacts of COVID-19 on urban air quality, the intra-city variabilities are thought to be further influenced by the sociological and geographical characteristics of the localities within each city. Liu et al. (2021) investigated the spatiotemporal impacts of COVID-19 on air quality in California, showing a decreasing NO_2_ trend at a location near a power plant but an increasing NO_2_ trend in a residential area. Han et al. (2020) showed a large decrease in the PM_2.5_ concentration at the city center of Seoul during the period of social distancing. Intra-city comparisons of air quality can also provide essential information for assessing the neighborhood-scale health effects of air pollutants (Mateos et al. 2018). For instance, Sasidharan et al. (2020) found an association between a region’s vulnerability to COVID-19 and the local air quality. As intra-city variation of urban air quality is often a major concern for urban residents, now in addition to the COVID-19 pandemic situation, a monitoring-based intra-city analysis of the spatial distribution of air pollutants is needed.

In this study, we investigate the inter- and intra-city variabilities in the PM_2.5_ concentration changes under COVID-19 social distancing in seven major cities of South Korea. The different PM_2.5_ emission amounts and meteorological conditions in these cities are expected to have a diversity of effects on the ways in which urban air quality can change under the influence of social distancing. In addition to the inter-city comparisons, intra-city comparisons, taking advantage of the densely distributed air quality monitoring stations, can offer a unique insight into the effects of social distancing.

## Data and methods

**Fig. 1:**
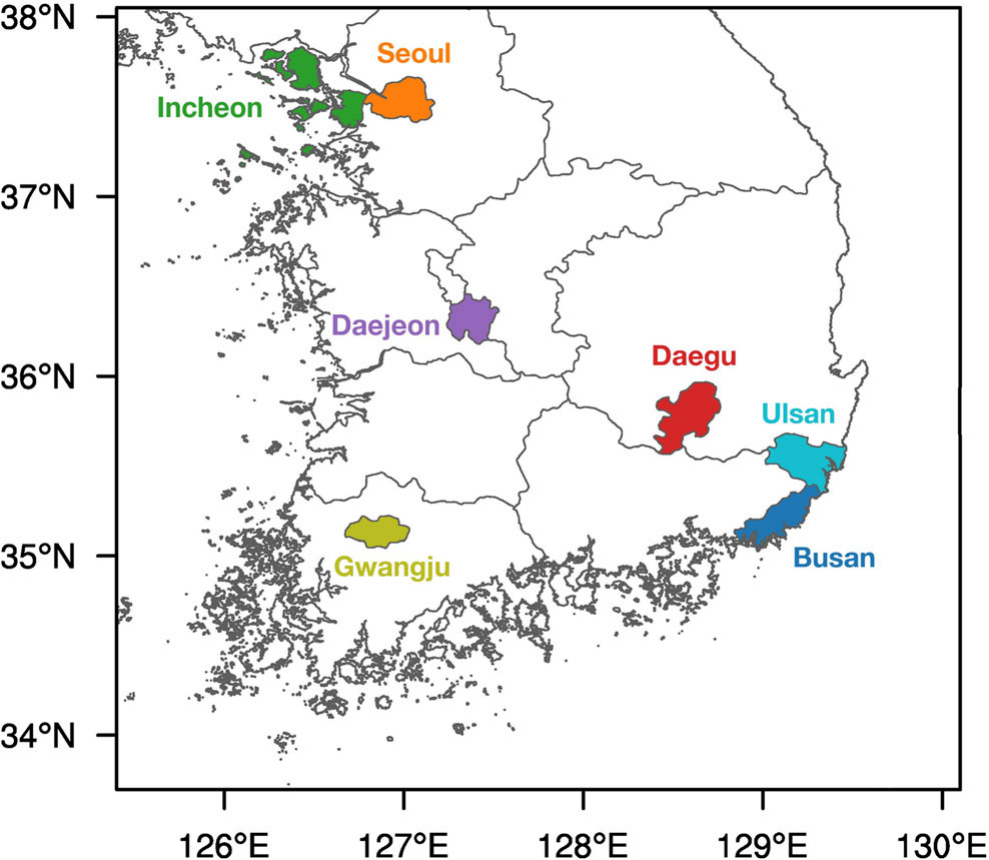
Map of South Korea showing administrative divisions including the seven major cities, Seoul, Busan, Incheon, Daegu, Daejeon, Gwangju, and Ulsan. Cities are indicated by fill colors and corresponding city names

The hourly averaged PM_2.5_ concentration data from air quality monitoring stations (AQMSs) in the seven major cities (Seoul, Busan, Incheon, Daegu, Daejeon, Gwangju, and Ulsan) of South Korea screened by Korea Environment Corporation (http://www.airkorea.or.kr) are used in this study. This study uses data from 25, 19, 20, 14, 10, 9, and 16 AQMSs in Seoul, Busan, Incheon, Daegu, Daejeon, Gwangju, and Ulsan, respectively. Figure 1 shows locations of the seven major cities. Seoul, Incheon, and Daejeon are located in the west-central region of the Korean Peninsula. The other cities are located in the southern region, with Busan, Daegu, and Ulsan in the southeast. Busan and Ulsan, the two southeastern coastal cities, share a border. Likewise, Seoul and Incheon are in close proximity of one another. The port city of Incheon consists of many islands, but its busiest city center is located on the mainland. According to the Korean Statistical Information Service (https://kosis.kr), as of July 2020, Seoul has the largest population (~9.7 million), followed by Busan (~3.4 million) and Incheon (~2.9 million). Ulsan has the smallest population (~1.1 million). Based on the emission data for the year 2017 provided by the National Air Pollutants Emission Service (http://airemiss.nier.go.kr) and the land area data from the Korean Statistical Information Service, the PM_2.5_ emission amounts per area tend to be larger in highly populated cities such as Seoul (4.8 ton km^−2^ year^−1^) and Incheon (5.4 ton km^−2^ year^−1^) than in other cities such as Daejeon (1.2 ton km^−2^ year^−1^) and Gwangju (1.3 ton km^−2^ year^−1^). Despite having the smallest population among the seven cities, Ulsan (3.0 ton km^−2^ year^−1^) also recorded a fairly large per-area PM_2.5_ emission amount in 2017 partly because of the many industrial complexes located there.

Following Han et al. (2020), 29 February 2020 is chosen as the starting date of social distancing. We analyze the PM_2.5_ concentration data in the periods of 30 days before (pre-SD, from 30 January to 28 February) and since (SD, from 29 February to 29 March) the starting date of social distancing. Discarding missing values, 160,153 measurement data (98.4% of total) are used in this study. Note that Daegu faced a particularly severe outbreak situation in mid-February and practiced stronger prevention measures against COVID-19 than the social distancing practiced in other cities (Yonhap News Agency 2020). This may have led to greater reductions in local emissions of air pollutants during the analysis periods in this city.

To examine the effects of meteorological variables on urban air quality during the analysis periods, 2-m air temperature and 10-m wind speed measured at a meteorological observatory equipped with the Automated Synoptic Observing System (ASOS) in each city are analyzed. The ASOS data are provided by the Korea Meteorological Administration (http://data.kma.go.kr). Time interval for the meteorological data is 1 h. Discarding 8 missing values, 30232 measurement data are used for the analysis.

## Results and discussion

### Changes in energy use and air quality under social distancing

City-wide energy uses directly reflect the societal changes during the COVID-19 pandemic. Table 1 compares the changes in monthly liquefied natural gas (LNG) supplies and electric power sales from 2019 to 2020 in the seven cities. The LNG supplies decreased by 3.2% and 8.4% for residential use and for other uses (including industrial, commercial, and public uses), respectively, from 2019 to 2020 in February. On the other hand, the LNG supplies increased by 3.0% only for residential use but dramatically decreased by 16.9% for other uses from 2019 to 2020 in March. The increases in the LNG supplies for residential use in March are the largest in Busan followed by Ulsan and Gwangju. Such increases in energy use at home during the period under social distancing are also shown in the electric power sales. The city-wide electric power sales in March increased by 7.6–10.5% for residential use from 2019 to 2020, while those for other uses were less variable. Although the energy use data do not give direct information on the change in PM_2.5_ emission, the above analysis provides evidence that there were notable changes in human activities under social distancing. The residents of the seven cities were more likely to stay at home under social distancing, leading to a suppression of such human activities that contribute to other non-residential energy uses.

**Table 1:** Monthly statistics of liquefied natural gas (LNG) supplies and electric power sales in the seven cities

	LNG supplies^a^ (10^6^ m^3^)				Electric power sales^b^ (GWh)			
	Residential use		Other uses		Residential use		Other uses	
	Feb.	Mar.	Feb.	Mar.	Feb.	Mar.	Feb.	Mar.
Seoul								
2019	446	315	216	164	1181	1034	2946	2549
2020	423	323	193	142	1193	1122	2845	2535
Rel. change (%)	−5.2	+2.6	−10.9	−13.8	+1.0	+8.5	−3.5	−0.5
Busan								
2019	100	76	67	68	421	364	1333	1288
2020	98	86	69	63	428	400	1354	1270
Rel. change (%)	−1.6	+13.0	+2.7	−7.7	+1.7	+10.0	+1.6	−1.4
Incheon								
2019	110	83	83	87	366	319	1662	1688
2020	105	85	72	69	372	348	1691	1650
Rel. change (%)	−4.8	+2.4	−13.4	−21.4	+1.6	+9.2	+1.7	−2.3
Daegu								
2019	96	86	43	37	296	258	1031	956
2020	97	82	43	37	300	284	1014	917
Rel. change (%)	+0.6	−4.3	−2.2	−1.9	+1.4	+10.1	−1.6	−4.1
Daejeon								
2019	56	45	38	37	179	155	636	605
2020	58	45	40	31	179	167	652	604
Rel. change (%)	+3.8	−0.7	+4.4	−16.1	+0.3	+7.6	+2.5	−0.1
Gwangju								
2019	52	43	26	25	181	159	560	523
2020	52	44	25	24	186	174	554	523
Rel. change (%)	−0.2	+3.2	−3.8	−3.8	+2.3	+9.1	−1.1	+0.0
Ulsan								
2019	48	34	160	188	139	120	2483	2689
2020	46	37	139	139	142	133	2566	2723
Rel. change (%)	−2.8	+8.9	−12.8	−25.8	+2.1	+10.5	+3.3	+1.3
Total								
2019	909	682	634	606	2763	2409	10651	10298
2020	880	703	581	504	2800	2628	10675	10223
Rel. change (%)	−3.2	+3.0	−8.4	−16.9	+1.3	+9.1	+0.2	−0.7

^a^Monthly data from the Korean City Gas Association (http://www.citygas.or.kr)

^b^Monthly data from the Korea Electric Power Corporation (KEPCO) (http://bigdata.kepco.co.kr)

**Fig. 2:**
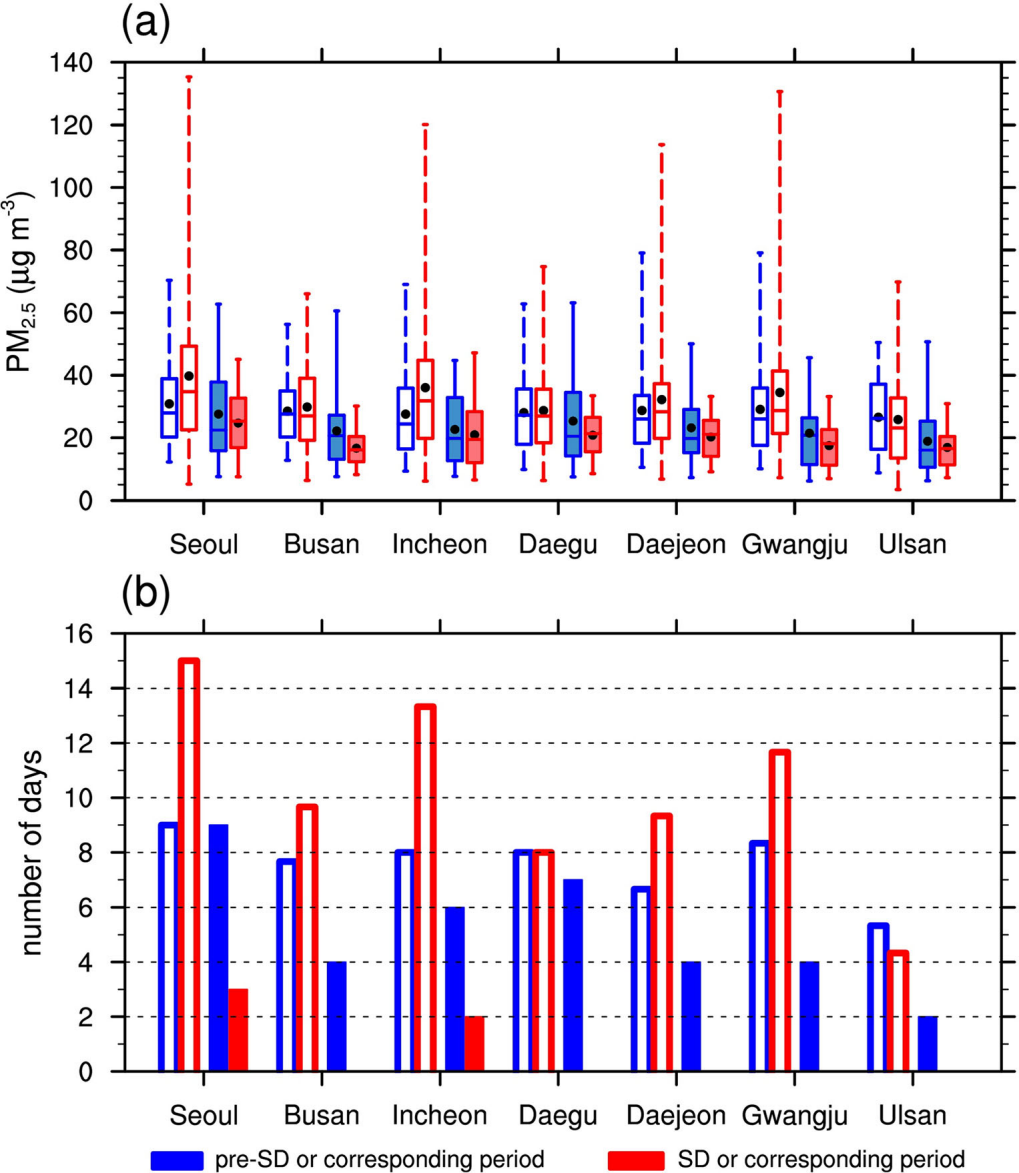
**a** Box-plot of the daily-mean PM_2.5_ concentrations in the seven cities during pre-SD (filled blue box) and SD (filled red box) and the periods corresponding to pre-SD (unfilled blue box) and SD (unfilled red box) in 2017–2019. The upper and lower quartiles are represented by the upper and lower edges of each box, respectively. The median and 30-day mean values are indicated by the center line and black dot inside each box, respectively. The whiskers above and below each box reach the maximum and minimum values, respectively. **b** Bar chart for the number of days with PM_2.5_ concentration exceeding 35.0 μg m^−3^ for each city during pre-SD (filled blue box) and SD (filled red box) and averaged over the periods corresponding to pre-SD (unfilled blue box) and SD (unfilled red box) in 2017–2019. The results for Seoul during pre-SD and SD are adapted from Han et al. (2020)

Changes from pre-SD to SD in the daily-mean PM_2.5_ concentrations and the number of days with “high” pollution levels (i.e., PM_2.5_ concentration exceeding 35.0 μg m^−3^) in the seven major cities of South Korea are presented and compared to the changes for the corresponding periods in the previous three years (2017–2019) in Fig. 2. The days with “high” pollution levels are determined following the same definition given in Han et al. (2020). Note that the results for Seoul in Fig. 2 are adapted from Han et al. (2020). From pre-SD to SD, the 30-day means and upper quartiles of the daily-mean PM_2.5_ concentrations decreased in all seven cities (Fig. 2(a)). The maxima also decreased in all cities except Incheon. In contrast, there were relatively small changes in the lower quartiles and the minima. This means that the temporal variability of PM_2.5_ concentration decreased during the period under social distancing. Decreases in PM_2.5_ concentrations under the COVID-19 prevention measures were also seen in other cities around the world (e.g., Bao and Zhang 2020; Ma and Kang 2020). The PM_2.5_ changes from pre-SD to SD were overall in the opposite direction to those in the previous years. From the period corresponding to pre-SD to the period corresponding to SD in 2017–2019, the 30-day means of PM_2.5_ concentrations increased in all cities except Ulsan and the upper quartiles of the daily-mean PM_2.5_ concentrations increased in all cities except Daegu and Ulsan. The effects of social distancing might have played a role in the unusual PM_2.5_ changes in 2020 compared to the previous years. From air quality model simulations, Kang et al. (2020) found that, with regard to the observed reduction of PM_2.5_ concentration in South Korea during the COVID-19 period, the effects of the reduction of anthropogenic emission were much greater than the effects of the changes in meteorological condition.

### Inter-city comparisons

The inter-city variability in the decreased amounts of the 30-day mean PM_2.5_ concentration was substantial. Table 2 lists for each city the decrease in the 30-day mean PM_2.5_ concentration from pre-SD to SD in terms of both the magnitude and the relative change. Among the seven cities, Busan experienced the most dramatic decrease (−24.9%) in the 30-day mean PM_2.5_ concentration, followed by Gwangju (−18.6%) and Daegu (−17.9%). In addition, Busan and Daegu showed the largest decreases in the upper quartiles (−25.1% and −23.2%, respectively) but no big changes in the lower quartiles, indicating that the decreases in the 30-day mean PM_2.5_ concentration in these two cities were mainly due to the smaller number of highly polluted days. By comparison, Incheon (−7.5%), Seoul (−10.4%), and Ulsan (−10.5%) experienced relatively small decreases in the 30-day mean PM_2.5_ concentration. It is surprising that the decrease in the 30-day mean PM_2.5_ concentration in Ulsan was much smaller than that in Busan despite these two cities being near each other geographically (Fig. 1). Comparisons between the pairs of neighboring cities (i.e., Seoul–Incheon and Busan–Ulsan) show that between two geographically adjacent cities, the city with a higher PM_2.5_ concentration in pre-SD is more likely to exhibit a larger decrease in PM_2.5_ concentration in SD.

**Table 2:** Difference (SD minus pre-SD) and relative change in the 30-day mean PM_2.5_ concentration averaged over all stations in each of the seven cities from pre-SD to SD

City	Difference (μg m^−3^) SD – pre-SD	Relative change (%) from pre-SD to SD
Seoul	−2.9	−10.4
Busan	−5.5	−24.9
Incheon	−1.7	−7.5
Daegu	−4.5	−17.9
Daejeon	−2.9	−12.7
Gwangju	−4.0	−18.6
Ulsan	−2.0	−10.5

The number of days with “high” levels of PM_2.5_ pollution substantially went down under social distancing in all seven cities (Fig. 2(b)). This decrease is in contrast with the increase in the corresponding periods in 2017–2019 in all cities except Daegu and Ulsan. The decrease in the number of days with “high” levels of PM_2.5_ pollution from pre-SD to SD is closely related to the decreases in the upper quartiles of PM_2.5_ concentration found in all seven cities as shown in Fig. 2(a). It is particularly notable that while Daegu had the second highest count of the days with “high” PM_2.5_ pollution levels during pre-SD, the number went down to zero during SD. Due to the severity of the COVID-19 outbreaks, Daegu was the only city in South Korea that practiced a much stronger restriction on human activity compared to social distancing practiced in other cities. Busan, Daejeon, Gwangju, and Ulsan also witnessed this vanishing of the days with “high” PM_2.5_ pollution levels during SD. In cities in the west-central region such as Seoul and Incheon, the number of days with “high” levels of PM_2.5_ pollution still remained non-zero during SD. This may reflect the possibility that westerly long-range (transboundary) transport of PM_2.5_, typical around this time of the year, also had a considerable influence on PM_2.5_ concentrations in these two cities during SD.

**Fig. 3:**
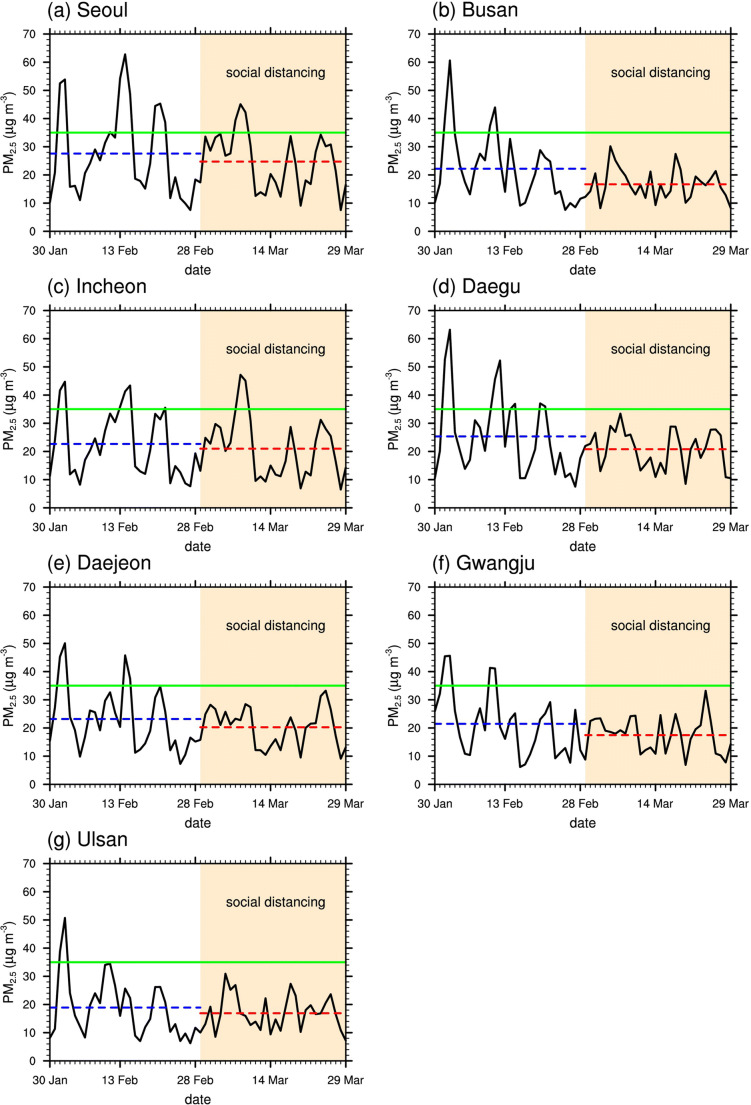
Time series of the daily-mean PM_2.5_ concentration during pre-SD and SD in **a** Seoul (adapted from Han et al. 2020), **b** Busan, **c** Incheon, **d** Daegu, **e** Daejeon, **f** Gwangju, and **g** Ulsan. The 30-day mean values for pre-SD and SD in each city are indicated by the blue and red horizontal dashed lines, respectively. The “high” level of PM_2.5_ concentration (35.0 μg m^−3^) is represented by the horizontal green solid line. The shaded areas represent SD, the period of social distancing

To further examine the inter-city differences in their responses to social distancing in terms of air quality, the time series of the daily-mean PM_2.5_ concentrations in the seven cities are compared in Fig. 3. The time series in Seoul is adapted from Han et al. (2020). The fluctuations in the PM_2.5_ concentration having a period of several days or more suggest their dependence on the changing meteorological conditions which can affect long-range transport and the degree of air stagnation. Cities that are geographically close to each other showed similar fluctuation patterns in PM_2.5_ concentration. For example, the cities located in the west-central region (Seoul, Incheon, and Daejeon) had higher peaks on 14–15 February than on 11 February, while the southeastern cities (Busan, Daegu, and Ulsan) had higher peaks on 10–12 February than on 14–15 February. These suggest that the fluctuations were caused by phenomena on a scale that is larger than city-scale.

There were noticeable decreases in the amplitudes of the PM_2.5_ concentration fluctuations from pre-SD to SD (Fig. 3). This is related to the decrease in the temporal variability of PM_2.5_ concentrations shown in Fig. 2. The reduced fluctuation amplitudes prevented many of the PM_2.5_ concentration peaks during SD from reaching the “high” level of PM_2.5_ pollution. One possible explanation for these reduced amplitudes is the effects of social distancing. Reductions in local emissions from motor vehicles (on-road mobile sources) due to social distancing may have flattened these PM_2.5_ concentration peaks. Busan’s pre-SD fluctuation pattern was similar to that in the neighboring city of Ulsan but with higher peaks. During SD, on the other hand, the peak values for Busan were similar to those for Ulsan. The above reasoning is also supported by the decrease in CO concentration, an indicator of vehicle emission, which was more prominent in Busan (−12.3%) than in Ulsan (−10.5%). Daegu, which showed the second largest decrease (in %) in the upper quartile of the daily-mean PM_2.5_ concentration (Fig. 2(a)), had the largest decrease in CO concentration (−28.6%) going from pre-SD to SD, which is also in line with Seo et al. (2020).

**Fig. 4:**
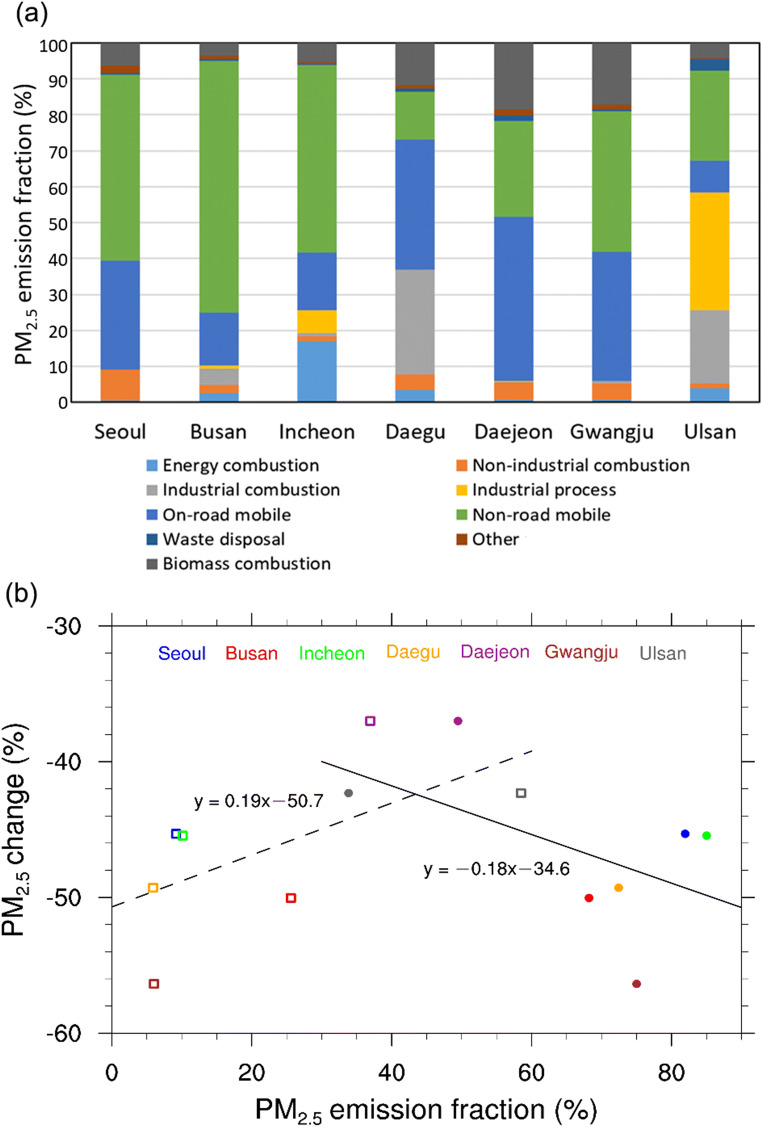
**a** PM_2.5_ emission fractions (in %) from major sources in the seven cities and **b** the scatter plot of the cumulative PM_2.5_ emission fractions (in %) for combustion and industrial process sources (open squares) and for mobile sources (closed circles) against the PM_2.5_ change ratios (in %) from the SD period compared to the corresponding period in 2019 in the seven cities. The dashed and solid lines indicate the linear regression trendlines for the scatters for combustion and industrial process sources (open squares) and those for mobile sources (closed circles), respectively

The relationship between the changes in PM_2.5_ concentration and the PM_2.5_ emission fractions in the seven cities found in Fig. 4 could quantitatively show the causality between urban air quality and local emission. Note that we calculated the change in PM_2.5_ concentration in Fig. 4(b) by subtracting the PM_2.5_ concentration for the 30-day period corresponding to SD in 2019 from that for SD in 2020 to exclude the effects of seasonality in PM_2.5_ emission. The annual PM_2.5_ emission data were estimated based on the year 2017 and were released in July 2020 by the National Air Pollutants Emission Service. The cumulative fraction in PM_2.5_ emission for combustion (energy, non-industrial, and industrial) and industrial process sources is the largest in Ulsan (58%), followed by Daegu (37%) and Incheon (26%). Gwangju (6%) and Daejeon (6%) have small fractions in PM_2.5_ emission for combustion and industrial process sources. On the other hand, the cumulative fraction in PM_2.5_ emission for mobile sources (traffic and construction machinery) is the largest in Busan (85%), followed by Seoul (82%) and Gwangju (75%). We have found that the fraction in PM_2.5_ emission for combustion and industrial process sources has a positive correlation (*R* = 0.62) with the change in PM_2.5_ concentration between SD in 2020 and the corresponding 30-day period in 2019. In contrast, the fraction in PM_2.5_ emission for mobile sources has a negative correlation (*R* = −0.54) with the change in PM_2.5_ concentration between SD in 2020 and the corresponding 30-day period in 2019. This implies that the reduction in PM_2.5_ concentration was substantial in cities that had large fractions in PM_2.5_ emission for mobile sources rather than for industrial process and other sources. The relationship between the fraction in PM_2.5_ emission and the reduction in PM_2.5_ concentration confirms the causality between local emission from mobile sources and urban air quality, particularly during the period when social distancing was practiced.

**Fig. 5:**
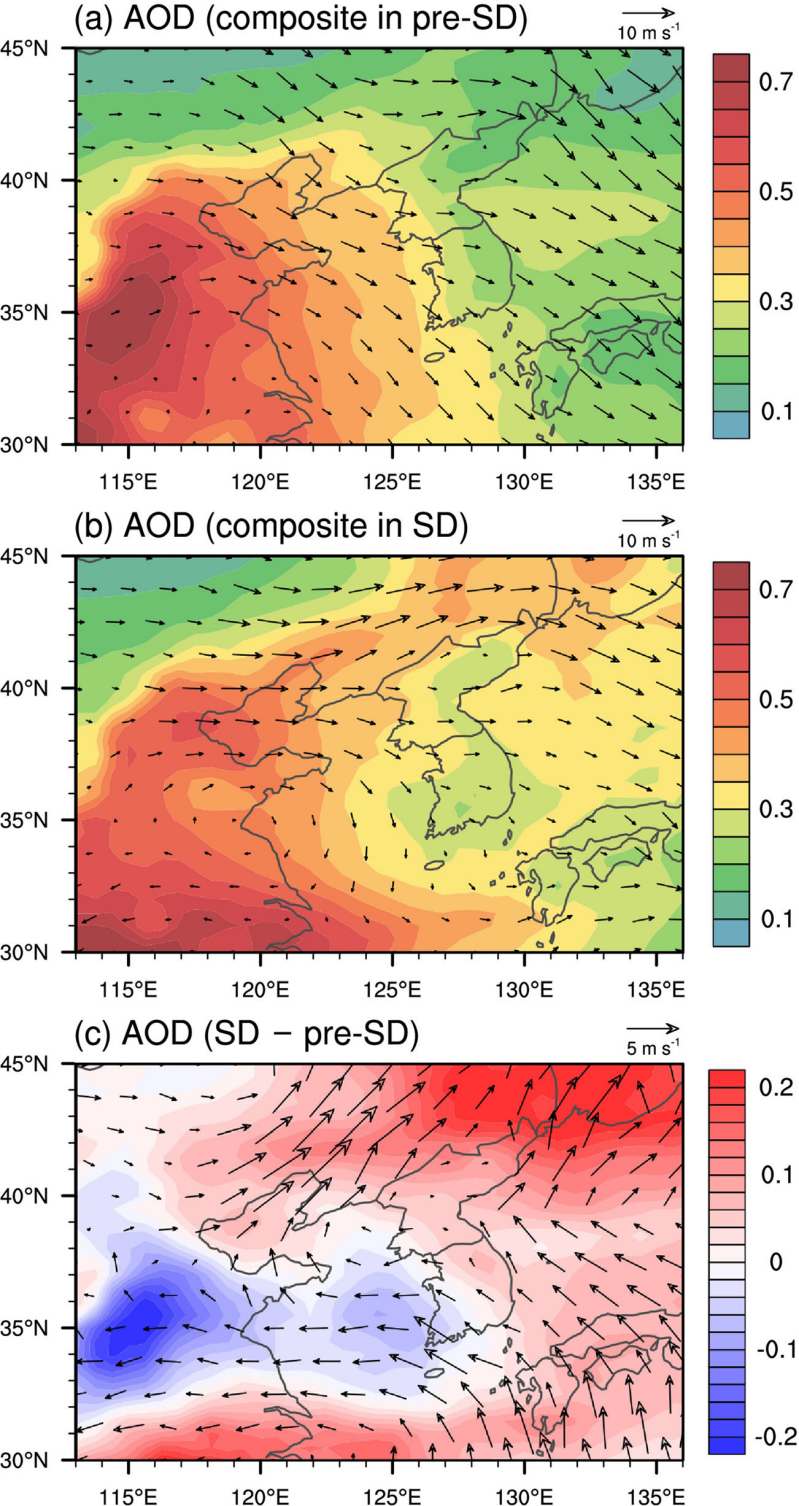
AOD and wind vector at the 900-hPa level averaged over the selected days in **a** pre-SD and **b** SD, and **c** differences in the averaged AOD and wind vector at the 900-hPa level from pre-SD to SD

Besides local emission, the changes in the PM_2.5_ concentration are also attributed to changes in meteorological conditions and long-range transport. Koo et al. (2020) noted that the changes in regional meteorology and the air quality improvement in China are largely responsible for the reduced aerosol concentration in South Korea under social distancing. Table 3 lists for each city the 30-day mean values of 2-m air temperature and 10-m wind speed for pre-SD and SD along with their differences (SD minus pre-SD). Note that air temperature is de-trended for calculation of correlation coefficients shown in the last column of Table 3 in order to exclude the seasonal rise of air temperature. All seven cities show positive correlations between de-trended air temperature and daily-mean PM_2.5_ concentration. The correlation coefficient between PM_2.5_ concentration and de-trended air temperature is higher in the cities in the west-central region (Seoul, Incheon, and Daejeon) than in the other cities. It seems that large-scale air temperature advection can produce the fluctuations in de-trended air temperature and also carry air pollutants depending on its direction (see Fig. 5). Wind speed is also an important meteorological variable because strong winds can act to enhance dispersion of air pollutants and decrease pollutant concentrations. Strong winds can also inhibit accumulation of aerosol precursors needed for secondary formation of PM_2.5_. During the analysis period, wind speed is negatively correlated with PM_2.5_ concentration. Busan experienced the second largest increase in the 30-day mean wind speed from pre-SD to SD (0.5 m s^−1^), which may have contributed to the substantial decrease in the 30-day mean PM_2.5_ concentration there.

**Table 3:** Thirty-day mean values of meteorological variables (2-m air temperature and 10-m wind speed) for pre-SD and SD averaged over all stations in each city. Correlation coefficients (*R*) between the daily-mean PM_2.5_ concentrations and the meteorological variables over the 60-day period. Temperature used to compute correlation coefficients is de-trended by subtracting the linear regression fit from the original data

	Pre-SD	SD	Difference SD – pre-SD	Correlation coefficient (*R*) against PM_2.5_
Seoul				
Temperature (°C)	2.5	7.5	5.0	0.46*
Wind speed (m s^−1^)	2.3	2.6	0.2	−0.36*
Busan				
Temperature (°C)	7.0	10.2	3.2	0.24
Wind speed (m s^−1^)	3.0	3.6	0.5	−0.33*
Incheon				
Temperature (°C)	2.5	6.9	4.4	0.49*
Wind speed (m s^−1^)	3.1	3.7	0.6	−0.43*
Daegu				
Temperature (°C)	4.9	9.1	4.2	0.35*
Wind speed (m s^−1^)	2.5	2.6	0.1	−0.45*
Daejeon				
Temperature (°C)	3.6	8.2	4.6	0.44*
Wind speed (m s^−1^)	1.3	1.6	0.3	−0.42*
Gwangju				
Temperature (°C)	5.1	8.7	3.6	0.35*
Wind speed (m s^−1^)	1.4	1.7	0.3	−0.40*
Ulsan				
Temperature (°C)	5.9	9.5	3.6	0.33*
Wind speed (m s^−1^)	2.2	2.3	0.1	−0.20

*Statistically significant (*p* < 0.05)

To assess the impacts of long-range transport and meteorological conditions, the AOD (aerosol optical depth) from the MERRA-2 data (Gelaro et al. 2017) are analyzed for selected days in pre-SD and SD such that the 900-hPa wind speed averaged over the seven South Korean cities is between 3 and 9 m s^−1^. In this way, we select the days with favorable conditions for the long-range transport of PM_2.5_ to strongly influence air quality in the South Korean cities. Note that the PM_2.5_ concentration averaged over the seven cities is 30.6% and 10.7% higher for the selected days than for the other days in pre-SD and SD, respectively. The 900-hPa wind speed is obtained from the ERA5 data (Hersbach et al. 2018) provided by the ECMWF (European Centre for Medium-Range Weather Forecast). This results in a total of 18 days out of the 30 days both in pre-SD (30, 31 January and 1, 2, 3, 4, 6, 7, 9, 10, 11, 13, 14, 15, 19, 26, 27, 28 February) and in SD (29 February and 1, 3, 5, 6, 7, 8, 9, 12, 13, 16, 18, 22, 24, 25, 27, 28, 29 March) for compositing the AOD and wind field data.

Figure 5 shows the AOD and the 900-hPa wind fields averaged over the selected days in pre-SD and SD. In pre-SD, the averaged wind field features northwesterlies over the Yellow Sea that transport aerosols to the South Korean cities (Fig. 5(a)). In SD, however, the wind direction over the Yellow Sea south of ~36°N changes from northwesterly to northerly (Fig. 5(b)). The wind speeds over the Yellow Sea also appear to be generally lower in SD than in pre-SD (Fig. 5(c)). Such wind field changes indicate that the synoptic weather patterns in SD may have been less favorable for the long-range transport of air pollutants to South Korea. In addition, there is a decrease in AOD over the region that lies between ~32–38°N and ~113–120°E, possibly caused by overall improvement of aerosol air quality there around this time period (Wang and Zhang 2020). The decrease in the AOD averaged over the selected days going from pre-SD to SD is particularly pronounced over a wide area over the southern part of the Yellow Sea, which also covers Daejeon and Gwangju (Fig. 5(c)). The increases in the magnitudes of relative changes in PM_2.5_ concentration were greater in Daejeon (−20.8% compared to −12.7% for 30-day means) and Gwangju (−28.8% compared to −18.6% for 30-day means) than in the other cities. The analysis results suggest that the reduced long-range transport of pollutants in SD might have affected the air quality in Daejeon and Gwangju more significantly than in the other cities.

The above results suggest that the inter-city variabilities are caused not only by the varying degrees of human activity level reductions in these cities in response to social distancing, but also by the differences among the cities in the contributions of long-range transport and meteorological conditions.

### Intra-city comparisons

**Fig. 6:**
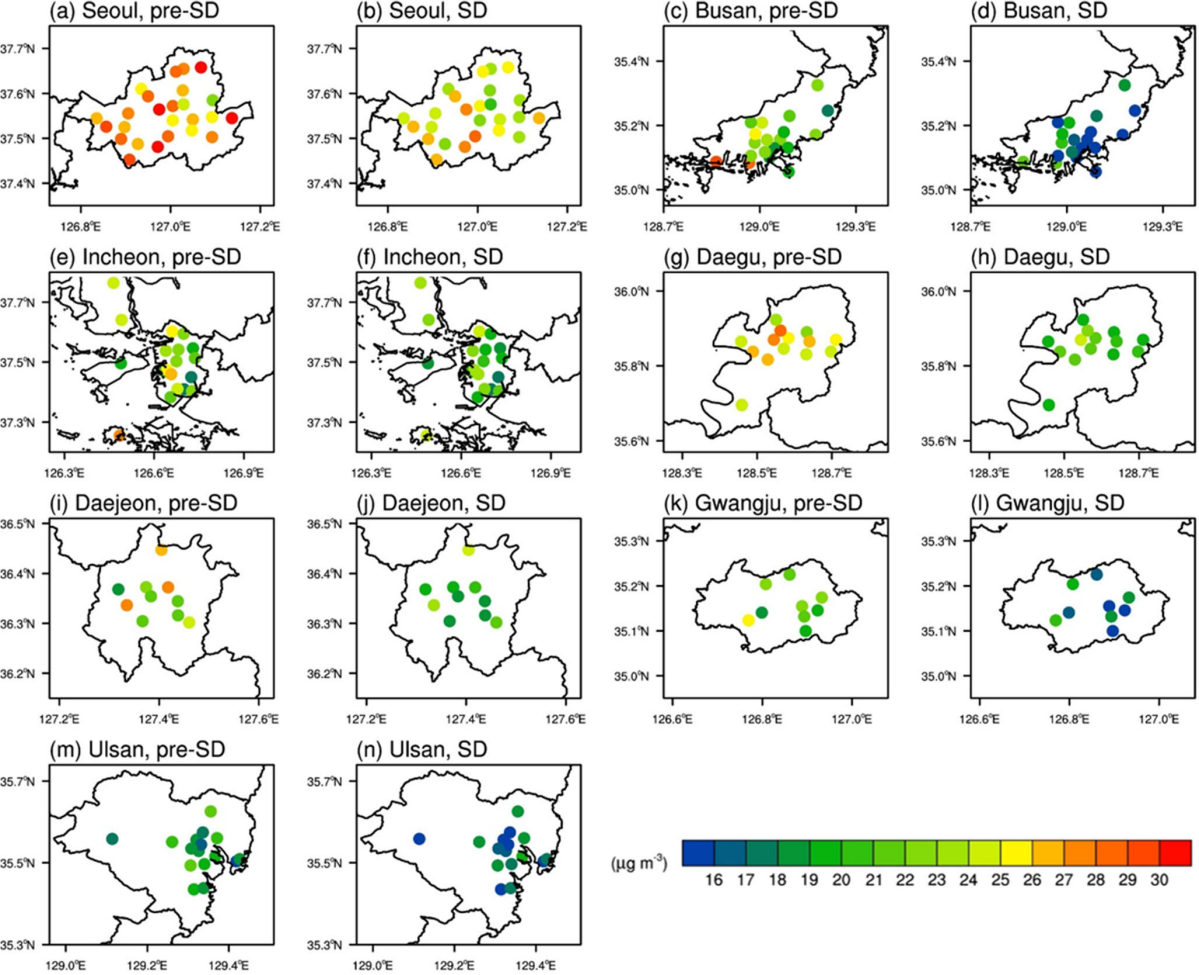
Spatial distributions of the 30-day mean PM_2.5_ concentrations for pre-SD and SD in **a**, **b** Seoul (adapted from Han et al. 2020), **c**, **d** Busan, **e**, **f** Incheon, **g**, **h** Daegu, **i**, **j** Daejeon, **k**, **l** Gwangju, and **m**, **n** Ulsan

In addition to the inter-city comparisons made so far using the quantities averaged over all AQMSs for each city, comparing the individual data from the AQMSs within each city can also provide important information characterizing the air quality changes under social distancing in that city. The spatial distributions of the PM_2.5_ concentrations at individual AQMSs averaged over the 30 days before and since the start of social distancing are shown in Fig. 6 for all seven major cities of South Korea. To quantify variations in the PM_2.5_ concentration distribution in each city, the standard deviation (*σ*) as well as mean (*μ*) of the 30-day mean PM_2.5_ concentrations among all AQMSs are computed for each city (Table 4). Entering SD, these intra-city standard deviation values decreased in all seven major cities of South Korea with an average decrease of ~12.9% from pre-SD to SD, meaning that there were reduced levels of spatial variation under social distancing. Indeed, comparing the spatial distributions between pre-SD and SD (Fig. 6) shows that most of the noticeably strong signals at individual AQMSs appear to have weakened under social distancing.

**Table 4:** Mean (*μ*) and standard deviation (*σ*) of the PM_2.5_ concentrations among the AQMSs in each city during pre-SD and SD. The highest, upper (Q3), and lower (Q1) quartiles, and lowest of the relative changes from pre-SD to SD in the 30-day mean PM_2.5_ concentration. The differences between the lower and upper quartiles (Q1 minus Q3) in percentage points (pp). Bold indicates the largest decreases among the seven cities for each category (Highest, Q3, Q1, and Lowest). Negative and positive signs are assigned for decreases and increases, respectively

	*μ* ± *σ* (μg m^−3^)	Relative change (%) from pre-SD to SD				Q1 – Q3 (pp)
		Highest	Q3	Q1	Lowest	
Seoul						
Pre-SD	27.6 ± 2.2	4.0	−8.5	−13.1	−18.7	−4.7
SD	24.7 ± 1.9					
Busan						
Pre-SD	22.2 ± 3.2	−10.3	−**22.6**	−**27.5**	−**33.5**	−4.9
SD	16.7 ± 2.9					
Incheon						
Pre-SD	22.7 ± 2.8	−1.8	−5.1	−10.4	−12.1	−5.3
SD	21.0 ± 2.2					
Daegu						
Pre-SD	25.3 ± 1.6	−12.1	−15.7	−19.5	−22.5	−3.8
SD	20.8 ± 1.4					
Daejeon						
Pre-SD	23.2 ± 3.0	3.5	−12.4	−14.1	−23.1	−1.7
SD	20.2 ± 2.3					
Gwangju						
Pre-SD	21.5 ± 2.1	−**13.8**	−14.3	−19.8	−28.6	−5.5
SD	17.5 ± 1.9					
Ulsan						
Pre-SD	18.9 ± 1.6	−0.7	−7.0	−14.0	−20.5	−7.0
SD	16.9 ± 1.6					

Table 4 also lists the relative change in the 30-day mean PM_2.5_ concentrations from pre-SD to SD for each city corresponding to the AQMSs with the highest and lowest of the relative changes in the city as well as the AQMSs exhibiting the relative changes at the upper and lower quartiles. Of the seven cities, Ulsan recorded the largest difference between the two stations at the lower and upper quartiles of the 30-day mean PM_2.5_ concentration. This might be partly attributed to the complex land-use distribution in this coastal industrial city of Ulsan. Compared to the other cities, this difference between the lower and upper quartiles was the smallest for Daejeon. There is a general tendency of the coastal cities (Ulsan, Busan, and Incheon) showing larger differences between the lower and upper quartiles than inland cities (Daejeon and Daegu). The coastal cities also experienced greater increases in the LNG supplies for residential use in March compared to the other inland cities (Table 1). This implies that, compared to the intra-city variabilities in Daejeon and Daegu, those in Ulsan, Busan, and Incheon were more strongly influenced by larger reduction in PM_2.5_ emission near intra-city sources (e.g., industrial complexes) due to social distancing. Among all AQMSs, the lowest value of the relative change (i.e., the largest decrease) in the 30-day mean PM_2.5_ concentration from pre-SD to SD was recorded at an AQMS in Busan. Note that not all AQMSs recorded decreases in the 30-day mean PM_2.5_ concentration from pre-SD to SD. In fact, a few AQMSs located far away from PM_2.5_ emission sources in Seoul and Daejeon showed slight increases in the 30-day mean PM_2.5_ concentration from pre-SD to SD.

It is notable that the maximum intra-city difference in the relative change (i.e., “Lowest” minus “Highest” in Table 4) in each city, ranging from −10.3 pp in Incheon and Daegu to −26.6 pp in Daejeon, is comparable to the maximum inter-city difference of −17.5 pp between Busan (−24.9%) and Incheon (−7.5%). This shows that there can be a significant variability among different locations in the same city in their responses to social distancing, sometimes surpassing even the inter-city variability among the cities in different parts of the country. Therefore, intra-city comparisons can also provide relevant information when it comes to assessing the effects of social distancing on local emission and urban air quality.

## Conclusion

Using the measurement data from the AQMSs, this study analyzed the changes in PM_2.5_ concentrations in the seven major cities of South Korea during the period of COVID-19 social distancing. The major influences of social distancing on PM_2.5_ concentration are twofold: (1) reductions in city-wide PM_2.5_ emissions, which show inter-city variations, and (2) reductions in the intra-city variations of PM_2.5_ concentration. The inter-city variations in the reductions in PM_2.5_ concentration were caused by both the differences among the cities in their PM_2.5_ emission fractions and the differences in how much long-range transport of air pollutants affected air quality in these cities. Meanwhile, the intra-city variability in response to social distancing was higher for coastal cities compared to inland cities.

The above results highlight the importance of intra-city analysis, in addition to inter-city analysis, in assessing the true impacts of social distancing on urban air quality. To overcome the difficulties in using measurement data alone for examining individual contributions of local emission, secondary formation, long-range transport, and meteorological conditions to PM_2.5_ changes, modeling studies using a high-resolution air quality model coupled with a high-resolution meteorological model with an urban canopy model (e.g., Ryu et al. 2011) that parameterizes various urban effects are needed. Such endeavors will also require an input of urban parameters that accurately reflect the intra-city differences of urban characteristics.

## Data Availability

The datasets generated during and/or analyzed during the current study are available from the corresponding author on reasonable request.
